# School-Based Approaches to Prevent Depression in Adolescents

**DOI:** 10.7759/cureus.13443

**Published:** 2021-02-19

**Authors:** Krishna Priya Bodicherla, Kaushal Shah, Romil Singh, Nkechi C Arinze, Gaurav Chaudhari

**Affiliations:** 1 Psychiatry, Sri Devaraj Urs Medical College, Kolar, IND; 2 Psychiatry, Griffin Memorial Hospital, Norman, USA; 3 Critical Care, Mayo Clinic, Rochester, USA; 4 Internal Medicine/Community Medicine, Mercer University School of Medicine, Macon, USA; 5 Psychiatry, Johns Hopkins Bloomberg School of Public Health, Baltimore, USA

**Keywords:** depression, adolescent, school mental health services, school health services, psychosocial intervention, cbt, preventive health services, intervention and psychotherapy for children and adolescents, cognitive behavioral therapy, psychodrama and group therapy

## Abstract

Depression is one of the highest prevalent mental illnesses and is one of the common illnesses that can have its onset during childhood or adolescence. It is estimated that up to 20% of children experience mental illness worldwide. Preventing the onset of depression in children and adolescents should be a vital public health goal that will improve public health and decrease health care costs. We reviewed literature that described school-based interventions to prevent the onset of depression, reduce the severity of depressive symptoms, and enhance global functioning in adolescents. Our research also provides strategies for school-based intervention programs that are mainly categorized into three main subtypes. We also discussed each subtype and its advantages and limitations. The goal is to bring the readers an understanding of the importance of preventing depression on a community level, beginning at schools.

## Introduction and background

Adolescence is when the foundation of mental, social, and emotional well-being is established. Depression often emerges for the first time during the teenage years. About 20% of children experience mental illness worldwide per estimation [1]. This critical phase of life is a potential challenge for adolescents to overcome influences of youth development strategies, social and emotional learning, behavioral modeling, violence, unintentional injury, substance use, nutrition, and obesity coupled with hormonal changes which might eventually manifest as depression and other mental health disorders [2-4].

Among other mental health issues, depression is considered the highest prevalent mental health disorder [5]. A nationally representative survey of 10,123 adolescents aged 13 to 18 years indicated that the lifetime prevalence of depression is 11% and 14% [6]. Adolescents with major depressive disorder (MDD) have risk factors like family medical history, mental health issues, underlying general negative affectivity, socio-economic disadvantage, and a history of abuse or other stressors. It is also well known that adolescents with a major depressive disorder are more likely to engage in risk-taking and thrill-seeking behaviors like substance use, attempted and completed suicide. Major depressive disorder is also reported to be associated with unstable peer and family relationships, poor school performance, and everyday functioning [7].

Preventing the onset of depression in children and adolescents is an advanced and vital public health goal with the potential to improve public health and decrease health care costs [8]. In support of this, there is an interest in developing programs aimed at preventing the onset of depression, with several studies being published in the last two decades. Many systematic reviews and meta-analyses have recently determined that there is evidence for beneficial effects of preventive strategies for depression in children and adolescents [5,9,10]. This paper is intended to provide an overview of the rationale for depression prevention in adolescents and the school-based approaches, with a particular focus on randomized controlled trials of school-based indicated depression prevention programs. 

For this review paper, we conducted a literature search on PubMed, PubMed Central, and Medline using the keywords 'Adolescent,' 'School Health Services,' and 'School Mental Health Services' in the context of 'Depression,' 'Preventive Health Services,' and 'Randomized Controlled Trial.' We screened abstracts that were found through this indexed search to identify the articles relevant to our objective, which centers on school-based intervention for depression in adolescents.

## Review

Given the impairing nature of depressive symptoms, it is crucial to prevent the new incidence of depression and reduce depressive symptoms as it adversely affects the overall quality of life and increases the risk of depressive episodes in the future. More than two-thirds of the younger population suffering from depression do not receive treatment [11]. The lack of approach, early diagnosis, and availability of specific treatment has given rise to interest in more widely developed preventive interventions, with schools offering a comfortable environment for such programs [12,13]. A study reported only 16% of youth receive treatment for mental health conditions, and out of them over 75% were reported being identified and treated at schools. A school setting that helps prevent depressive symptoms at a younger age has numerous advantages. It reduces the problems with recruitment of participants, convenient transportation or commuting, and the inclusion of a broader range of populations with varying levels of depressive symptoms [14,15].

Prevention of sub-diagnostic manifestations of depression in childhood was found to reduce the onset of depressive episodes in the future [16]. Also, the school staff and the administration providing a supportive role to the children at school aids in decreasing the psychosocial difficulties adolescents face within the classroom. However, schools also create a negative impact on the emotional health of children. The three main stress factors for children at school were being bullied by friends, heavy academic work, and difficulty in relationships with teachers [17]. Prevention strategies can also increase awareness and reduce the stigmatization among the children by identifying previously unidentified emotional problems in youth [18]. 

In addition to depression, these programs also decrease the prevalence of anxiety symptoms [4]. About one-third of children also develop conduct disorder in addition to depression leading to poor performance and low self-esteem in school [19]. These prevention strategies also help to control co-morbid conduct disorder along with depression. Several researchers also indicated that depressed youth show hostility to others in certain social situations. These children also face difficulties in showing improvement in their interpersonal relationships [20]. 

Types of school-based interventions

We reviewed several articles that described the different school-based interventions to prevent the onset of depression, reduce depressive symptoms, and improve adolescents' global functioning. The strategies used for school-based intervention programs implemented so far can be categorized into three main subtypes: universal, selective, and indicated [21,22]. Each subtype had its distinct advantages and limitations. 

The universal program aims to provide mental health services to all the students to promote overall well-being. The selective programs are focused on individuals at an increased risk of developing depression due to any particular risk factor such as trauma, suicide in the family, the divorce of parents, etc. The indicated programs are directed towards those students who demonstrate early symptoms of depression [21,23]. 

Some of the universal programs that have been widely discussed have their relative advantages and disadvantages [24-29]. When it comes to selecting one of the three types of school-based interventions for implementation in the school, the school administration is observed to lean towards the universal programs to avoid the stigma and time constraints that are associated with the other programs [30]. It is one of the limitations in implementing an indicated intervention, besides the limited availability of resources [31]. The universal approaches are known to have limited treatment effects, and validity is questionable in terms of children who do not have mental health issues [13].

A limiting factor for the indicated interventions can be the correct diagnosis of children at risk and the limited population impact. There have been instances where delivering these indicated interventions have had practical difficulties [32]. In terms of finding a place where it is possible to reach out to most youth and enable a coordinated interaction over a period of time, school is one of the best settings. Often mental health promotion initiatives have to face obstacles like stigma, time constraints, suitable sites, etc., which can be significantly reduced in a school setting [15]. As the school is considered a place for learning, it provides an ideal environment where adolescents can acquire new life skills. Schools are a promising setting for cognitive behavioral therapy (CBT) based interventions. In theory, youth struggling with depression may be more likely to engage in the prevention program because they are more motivated to change. It may also be easier to acquire intervention skills when they are applied to current symptoms [33]. Findings from several studies have shown a significantly increased risk of developing major depression in adolescents with sub-clinical depression over two years [34,35]. It was meta-analyzed that the targeted interventions on high-risk youth yield better results than those obtained from the universal programs on the general population [5,30]. Other reasons for selecting this subpopulation are their better response rate to the cognitive-behavioral interventions and the association of depressive symptoms with the onset of major depression [7,34,36-38]. Cognitive-behavioral therapy is proven to be effective in reducing symptoms in universal trials and also in targeted high-risk youth group trials [35,39-45].

Findings and recommendations

We yielded and reviewed four papers on randomized controlled school-based trials to prevent depression in adolescents [35,42,43,45]. The first two papers were based on the same study, the second being a follow-up study of the previous one. The trials in all four studies reviewed were conducted in a very systematic manner. The correct sample size was ensured in all studies. The recruitment, screening, and diagnosis were made using standard measurement tools, and a good follow up was ensured to track the results of the interventions. Important factors like past and current medical history, socio-economic backgrounds, etc., were considered while analyzing the results. The interventions were performed either through trained psychologists or students of psychology, or teachers after being given appropriate training. The average duration was of an hour, and the average number of sessions over the studies was six. A summary of the trials is described in Table 1. 

**Table 1:** Randomized control studies of indicated school-based early intervention programs for depression CB: cognitive behavior; BA: bachelor's degree; N: sample population size; *: based on the follow-up data of the same study

Trial	Participants	Age of Participants	Average Age	N	Content of Intervention and Control Program	Program Leader	Number of Sessions	Follow-Up Duration
Rohde et al., 2014 [43]	Adolescents	13 to 19 years	15.5	378	CB group, minimal contact CB bibliotherapy, or educational brochure control	BA Psychology/Equal training	6	Post-test, 6 months
Rohde et al., 2015* [45]								1 year, 2 year
Stice et al., 2010 [42]	Adolescents	14 to 19 years	15.6	341	Group CB intervention, group supportive expressive intervention, CB bibliotherapy, or educational brochure control condition	Psychology graduate and undergraduate students	6	Pre-test, Post-test, 6 months, 1 year, 2 years
Clarke et al., 1995 [35]	Adolescents	14 to 19 years	15.3	172	CB and usual care	Trained caregivers	15	Post-test, 6 months, 12 months

It was uniformly observed in all the studies that the CBT intervention resulted in a statistically significant reduction in the onset of MDD in the study population compared to the control groups. This observation is encouraging and confirms the finding of the previous study that advocated the prevention measures, which are more significant than "treatment effects" for youth with subthreshold symptoms [30]. Participants who received CBT showed a more significant decrease in depressive symptoms than the bibliotherapy participants, but not compared to supportive-expressive participants over a follow-up period from one to two years. Regarding the third aim of improving secondary outcomes, no significant results were obtained in any of the studies. 

These results are also very encouraging, considering that real-world therapists carried them out. They lend credibility to the school-based approach of developing indicated prevention trials targeting adolescents with subclinical depression. These findings are also significant because there have been very few trials at the school level, which have made a comparison between the CBT intervention methods with the existing alternative methods or placebo. This comparison is essential if one has to promote this approach as efficacious or make a strong case favoring the indicated prevention approach over the universal approach. Preventive efforts made at an early stage of child development effectively prevent psychological morbidity and promote good mental health in school-going children. It also potentially saves the health care expenditure that would otherwise be incurred in the treatment and rehabilitation of adolescents suffering from the onset of major depressive disorder. 

Although the interventions reduced the symptoms of depression in the short term in all the studies, the effectiveness was gradually lost throughout follow-up, demonstrating a regression to mean effect. It encourages us to develop a long-term strategy to maintain or boost the intervention's impact utilizing the stable school infrastructure. A part of the academic workforce could be trained to become teacher program leaders and deliver this intervention on a constant and long-term basis. It is also essential to consider the ease and dissemination of the intervention strategies to enable its sustainability over the long term and attain maximal impact. Methods like CBT bibliotherapy look promising in this measure from the reviewed literature. These findings should be researched further to implement mass-level dissemination at the school level. 

The unprecedented coronavirus pandemic poses a more significant challenge and risk of causing mental disorders amongst people of all age groups, including children [46]. Due to the disruption of the normal school routine, children may develop anxiety, depression, mood disorders, and other mental illnesses. As found in recent studies, these risk factors can be remediated through psychotherapy and counseling services to support the well-being of student's mental health [47]. These services, including CBT, can be rendered through telepsychiatry by licensed professionals and healthcare workers [47,48]. It is of paramount importance that all children in psychological need receive immediate and timely care [47,49]. The government, local authorities, and school authorities must ensure adequate professional resources available to support the mental health need either through traditional methods or telemedicine [49,50].

An interesting thing about the method of conducting these trials was the smaller number of sessions and the focus on some selective components in these sessions, which produced better results than the previously conducted similar interventions that contained more than 12 sessions. It could be followed up with a cost-benefit analysis to enable the appreciation of this strategy in a better way.

Limitations

It would be worth mentioning a few limitations which came across reviewing the literature, addressing which could boost the initiative of researching and promoting school-based intervention strategies in a big way. The measurement of the outcomes was through the self-assessment tools given to the study participants. The sole reliance on adolescents' data can also reduce sensitivity and generability. An ideal setup would be to train the school staff to interact with the students and screen a wider group of students with subclinical depression. The distribution of the races and ethnicity in the study population was representative of the larger sample, but their sample sizes were not large enough to comment on the interventions' effectiveness across all individual racial and ethnic groups.

## Conclusions

In summary, the reviewed literature suggests that depression prevention has made good progress in the past few years, proving feasible and useful on a small scale. The next step is to emphasize large-scale dissemination and implementation of school-based depression prevention strategies. The school-based initiative for indicated intervention to prevent depression in adolescents is one of the most promising mental health avenues today. Additional effort and resources should be focused in this direction to achieve the ultimate goal of reducing the prevalence of depression in the youth. Thus, we recommended that more vigorous scientific research is carried out in the future. This research could benefit from longer durations of follow-up and the study of effect modifiers and moderators. Future research could also benefit from more accurate diagnostic methods, increasing the sample size, and analyzing the moderating effects of various determinants. Thorough cost-benefit analyses must be done to formulate effective and quality policies and access the funds required for such projects.
